# Reduced Prefrontal Short—Latency Afferent Inhibition in Older Adults and Its Relation to Executive Function: A TMS-EEG Study

**DOI:** 10.3389/fnagi.2017.00119

**Published:** 2017-05-02

**Authors:** Yoshihiro Noda, Reza Zomorrodi, Felicity Backhouse, Robin F. H. Cash, Mera S. Barr, Tarek K. Rajji, Robert Chen, Zafiris J. Daskalakis, Daniel M. Blumberger

**Affiliations:** ^1^Temerty Centre for Therapeutic Brain Intervention, Centre for Addiction and Mental HealthToronto, ON, Canada; ^2^Department of Psychiatry, University of TorontoToronto, ON, Canada; ^3^Division of Neurology, Division of Brain, Imaging and Behaviour – Systems Neuroscience, Department of Medicine, Krembil Research Institute, University Health Network, University of TorontoToronto, ON, Canada; ^4^Monash Alfred Psychiatry Research Centre, Central Clinical School, Monash University, The AlfredMelbourne, VIC, Australia; ^5^Centre for Addiction and Mental Health, Campbell Family Mental Health Research InstituteToronto, ON, Canada

**Keywords:** TMS-EEG, short-latency afferent inhibition, age-related changes, cognition, dorsolateral prefrontal cortex

## Abstract

Combining transcranial magnetic stimulation (TMS) with electroencephalography (EEG) allows for the assessment of various neurophysiological processes in the human cortex. One of these paradigms, short-latency afferent inhibition (SAI), is thought to be a sensitive measure of cholinergic activity. In a previous study, we demonstrated the temporal pattern of this paradigm from both the motor (M1) and dorsolateral prefrontal cortex (DLPFC) using simultaneous TMS–EEG recording. The SAI paradigm led to marked modulations at N100. In this study, we aimed to investigate the age-related effects on TMS-evoked potentials (TEPs) with the SAI from M1 and the DLPFC in younger (18–59 years old) and older (≥60 years old) participants. Older participants showed significantly lower N100 modulation in M1–SAI as well as DLPFC–SAI compared to the younger participants. Furthermore, the modulation of N100 by DLPFC–SAI in the older participants correlated with executive function as measured with the Trail making test. This paradigm has the potential to non-invasively identify cholinergic changes in cortical regions related to cognition in older participants.

## Introduction

Transcranial magnetic stimulation (TMS) allows for the non-invasive measurement of various inhibitory and excitatory processes in the human brain (Hallett, 2011). Different TMS paradigms produce distinct inhibitory responses in cortical activity, and TMS paradigms administered sequentially produce unique neurophysiological interactions (Chen, 2004; Ni et al., 2011). One such paradigm involves median nerve stimulation (MNS) followed by a single TMS pulse to the motor cortex (M1) approximately 20 ms later. The MNS followed by the inter-stimulus interval (ISI) of 20 ms produces a marked reduction of motor-evoked potentials (MEP) from a single TMS pulse (Classen et al., 2000; Tokimura et al., 2000), and has been termed “short-latency afferent inhibition” (SAI) (Sailer et al., 2003).

SAI can be modulated by acetylcholine agonists and anticholinergic agents, implying a direct role of cholinergic activity in the SAI cortical response (Di Lazzaro et al., 2000, 2002, 2005b). The muscarinic acetylcholine receptor antagonist scopolamine decreases SAI in healthy participants (Di Lazzaro et al., 2000). Moreover, lorazepam reduces (Di Lazzaro et al., 2005a) and diazepam slightly increases (Di Lazzaro et al., 2005b) SAI, suggesting that gamma-aminobutyric acid (GABA)_A_ receptor-mediated inhibitory circuits are also involved in this neurophysiological response.

SAI in M1 is thought to involve contributions from S1 through an indirect cortico-cortical projection (Cash et al., 2015), as well as a direct thalamocortical projection to M1. Clinically it is impossible to distinguish the effects of both contributions (Ferreri et al., 2012). Similarly, SAI in the DLPFC is also speculated to involve the contributions from S1 and thalamocortical projection to DLPFC. Indeed, the inter-stimulus interval of M1-SAI has been shown to occur as early as N20+0 ms (Tokimura et al., 2000; Fischer and Orth, 2011), while the optimal interstimulus interval of DLPFC-SAI is approximately N20+4 ms (Noda et al., 2016). Therefore, we can generalize that SAI in M1 and DLPFC are significantly mediated by both of cortico-cortical and thalamocortical projections. This is strongly supported by the finding that they display a shared electrophysiological signature in both domains, indicating that they are mediated by similar mechanisms.

The role of cholinergic activity in cognitive function has been well established (Kopelman, 1986; Everitt and Robbins, 1997; Erskine et al., 2004). The presence of a relationship between cognitive deficits and reduced SAI response supports the notion that SAI is a direct measure of cholinergic activity. Previous studies have demonstrated that SAI is significantly diminished in patients with central cholinergic deficit such as Alzheimer's disease (Di Lazzaro et al., 2002). Furthermore, these SAI impairments were restored by the administration of acetylcholinesterase inhibitors rivastigmine (Di Lazzaro et al., 2002, 2005b) and donepezil (Nardone et al., 2012). Additionally, the amount of SAI decreases with age (Young-Bernier et al., 2012). Moreover, the dorsolateral prefrontal cortex (DLPFC) has been reported to be closely involved in the cholinergic-mediated cognitive function in monkey studies (Croxson et al., 2011; Yang et al., 2013). Thus, taken together, cholinergic function varies with age and brain region, supporting the role of cholinergic function in cognitive changes that occur with aging and neurodegeneration.

Previous TMS–electroencephalography (EEG) studies have demonstrated that the SAI paradigm at M1 induces significant modulation of N100 of TMS-evoked potential (TEP) component in the midline central area (Bikmullina et al., 2009; Ferreri et al., 2012), and showed the N100 TEP component also correlated with attenuation of motor–evoked potentials (MEP) in SAI (Bikmullina et al., 2009). TMS can be combined with EEG to study cortical regions outside M1. Our previous study demonstrated that the SAI protocol in M1 induced significant TEP increases at N45, N100, and P180 at an ISI of N20+2 ms over the left central region of interest (ROI), whereas SAI in the DLPFC induced significant attenuation of P60 TEP and increase of N100 TEP at an ISI of N20+4 ms over the left frontal ROI (Noda et al., 2016). Additionally, we replicated the correlation between N100 TEP and SAI–MEP changes at M1 (Bikmullina et al., 2009). Our results demonstrated that the SAI response exists in the DLPFC, and may share common mechanisms of M1–SAI (Noda et al., 2016).

Prior studies have investigated the effect of SAI on MEP in older subjects compared with younger subjects (Degardin et al., 2011; Young-Bernier et al., 2012, 2014, 2015). These studies other than one (Degardin et al., 2011) demonstrated that SAI is reduced in older subjects (Hedges's *g* = 0.778, *p* < 0.0001) (Bhandari et al., 2016). However, there have been no studies that have investigated the SAI effects on TMS-evoked potentials (TEPs) in M1 or the DLPFC in older adults. Thus, the present study aimed to investigate the modulatory effects of SAI paradigm on TEP in both M1 and the DLPFC in older adults compared to younger adults. Based on previous work (Young-Bernier et al., 2012; Noda et al., 2016), we anticipated that N100 TEP component would be significantly altered with SAI administered to M1 and the DLPFC in the older compared to the younger participants. We also sought to examine whether there are any associations between the modulations of SAI–TEP and cognitive measures in the older participants.

## Methods

### Participants

Twelve younger (6 female, mean age ± SD; 39 ± 12 years, 22–57 years) and 12 older (6 female, mean age ± SD; 72 ± 9 years, 64–92 years) individuals participated in the present study. All participants were right-handed. In addition, there was no significant age difference (t_22_ = −0.270, *p* = 0.789) between female (54 ± 21 years) and male (56 ± 19 years) participants for all participants. Participants over 18 years were eligible to participate in this study if they met the following criteria: (i) no history of neurological disorders including seizure or stroke, (ii) no history of neuropsychiatric disorders, (iii) normal cognitive function, (iv) no history of alcohol or other drug abuse/dependence, and (v) did not smoke, use recreational substances or prescription medications. All participants were screened with either the Structured Clinical Interview for DSM–IV Axis I Disorders or the Mini-International Neuropsychiatric Interview (Sheehan et al., 1998) prior to study participation to exclude a history of psychiatric illness. The study was reviewed and approved by the Ethics Committee of the Centre for Addiction and Mental Health with written informed consent from all subjects, and was carried out in accordance with the recommendations of “*Non-invasive electrical and magnetic stimulation of the brain, spinal cord, roots and peripheral nerves: Basic principles and procedures for routine clinical and research application. An updated report from an International Federation of Clinical Neurophysiology Committee.”* All subjects gave written informed consent in accordance with the Declaration of Helsinki. The same procedures and analyses in the younger participants were applied in the older participants in this study.

### TMS procedure and EMG measure

Monophasic TMS pulses were administered to M1 on the left hemisphere using a 70 mm figure–of–eight coil, and two Magstim 200 stimulators (Magstim Company Ltd., UK) connected via a Bistim module. MEP data were collected using the commercially available software, Signal (Cambridge Electronics, UK). Participants were seated in a chair and instructed to relax and keep their eyes open. Surface electromyography (EMG) was recorded from Ag/AgCl electrodes placed over the belly of the first dorsal interosseous muscle in the right hand.

### SAI procedure

The SAI paradigm was administered using standard methods from the literature (Tokimura et al., 2000; Noda et al., 2016). Specifically, the median nerve was stimulated at the right wrist using a standard bar electrode, with a cathode positioned proximally using a constant current stimulator (Digitimer model DS7A, Digitimer Ltd., UK). It is noted that we chose to stimulate the median nerve since there is a relatively low specificity of peripheral nerve stimulation in terms of the strength of SAI (Cash et al., 2015). The conditioning MNS intensity (pulse width 200 μs) was adjusted to three times the sensory threshold. For the SAI paradigm, TMS was performed over the motor hotspot of the first dorsal interosseous muscle at an intensity that evoked a 1mV response in MEP amplitude peak–to–peak. SAI was delivered at the MNS–TMS ISIs relative to the somatosensory evoked potential (SSEP) at N20 (Fischer and Orth, 2011; Ferreri et al., 2012; Noda et al., 2016). To obtain the individual N20, SSEP were recorded before starting the SAI protocol (200 stimuli delivered at 3 Hz and 3 times sensory threshold). SSEP N20 was calculated using Neuroscan software (Compumedics Neuroscan) based on previously published method (Noda et al., 2016). Following to our previous method, we applied the ISI of N20+2 ms for the M1–SAI paradigm while we used the ISI of N20+4 ms for the DLPFC–SAI paradigm (Noda et al., 2016). TMS was applied to F5 electrode site in the DLPFC–SAI following previously published methodology (Rusjan et al., 2010). The inter-stimulus interval of the SAI protocol was 5 s and the number of TMS–EEG trials for each condition was 100 times (i.e., 200 trials in total for both M1 and DLPFC–SAI, respectively). Furthermore, the order of single test pulse (TS) or conditioned-test pulse (SAI), as well as the order of M1 or DLPFC stimulation site was randomized to avoid potential order and cumulative effects on MEP and TEP (Pellicciari et al., 2016). EMG measures of SAI (i.e., MEP–SAI) were calculated as a ratio of conditioned MEP amplitude (i.e., SAI) divided by the 1 mV peak–to–peak MEP amplitude (i.e., TS); that is, [conditioned MEP (SAI)]/[unconditioned MEP (TS)].

In the present study, we analyzed TEP modulations by SAI paradigm primarily with a ratio of TEP amplitude change (i.e., SAI/TS), and secondary with a TEP amplitude subtraction method (i.e., SAI – TS). The results analyzed in a ratio method are presented in figures and results analyzed in a subtraction method are demonstrated in Supplementary Figures.

### EEG recording and pre-processing

A Synamps 2/RT 64-channel EEG system and a Quik-Cap Electrode Placement System (Compumedics Neuroscan, Australia) were used to record cortical activity. All electrodes were referenced to an electrode placed on the vertex positioned electrode. EEG signals were recorded at DC at 20 kHz sampling rate and with a low pass filter of 200 Hz. EEG data were processed offline using MATLAB (MathWorks, Natick, MA). All data were down–sampled to 1,000 Hz for analyses.

### SAI–TEP data analysis

SAI–TEP data were analyzed based on the method in our previous study (Noda et al., 2016). The continuous EEG time series were sectioned to include data from −1,000 ms before to 2,000 ms after the TMS pulse and then baseline-corrected with respect to the pre-stimulus interval −500 to −110 ms. TMS artifact 10 ms after the TMS pulse was removed during data cleaning. The data was visually inspected, and trials and channels that were highly contaminated with noise (muscle activity, electrode artifacts) were subsequently removed from analysis. More than 80% of trials and 95% of channels remained after artifact removal. Before applying the independent component analysis cleaning method, each condition of EEG data was concatenated to avoid the bias of the independent component analysis (ICA) cleaning process. Then, the ICA (an automated version of the infomax ICA algorithm in the EEGLAB toolbox) (Bell and Sejnowski, 1995; Makeig et al., 1997; Delorme and Makeig, 2004) was applied to remove eye–related artifacts (blinks and eye movements), the remaining muscle artifacts, as well as the TMS-related decay artifacts immediately after the TMS pulse. Following the ICA, the Butterworth, zero-phase shift 1–55 Hz band pass filter (24 dB/Oct) and notch filter were applied. Finally, data was re-referenced to the average for further analyses. Of note, SAI–TEP values were obtained after subtracting the SSEP trace from the TEP individually for each participant, in accordance with our previous study evaluating SAI with TMS–EEG (Noda et al., 2016). Furthermore, we detected TEP values by identifying the peaks (i.e., P30, P60, and P180) and troughs (i.e., N45 and N100) individually. In addition, to evaluate the SAI effect on TEPs, we calculated the ratio for each TEP component in the M1–SAI and DLPFC–SAI, which represents the degree of modulation by SAI paradigm, as follows: Modulation of TEP = conditioned TEP (SAI) /unconditioned TEP (TS).

### Cognitive assessment

The Wechsler Test of Adult Reading (WTAR) (Holdnack, 2001), the Repeatable Battery for the Assessment of Neuropsychological Status (RBANS) (Howieson et al., 2004), the Stroop test (Howieson et al., 2004), and the Trail Making Test (TMT) Parts A & B (Bowie and Harvey, 2006) were performed in the older participants. The WTAR estimates a degree of intellectual functioning prior to the onset of neuropsychiatric or neurological disorder (Holdnack, 2001). The RBANS assesses five domains of cognition as follows: immediate memory, visuospatial and constructional memory, language, attention, and delayed memory (Howieson et al., 2004). The Stroop test measures some aspects of executive functioning such as selective attention, cognitive flexibility, inhibition, self-regulation capacity, and processing speed (Howieson et al., 2004). The TMT evaluates general executive functioning such as visual search speed, scanning, task switching, processing speed, and mental flexibility (Bowie and Harvey, 2006).

### Statistical analysis

SPSS version 19.0 was used for statistical analysis. Based on our previous findings (Noda et al., 2016), we have focused on the left central area as a ROI for M1–SAI analysis and the left frontal area as a ROI for the DLPFC–SAI analysis in the present study (see Supplementary Figure 1).

First, to examine the degree of modulation of TEPs (i.e., P30, N45, P60, N100, and P180) by the M1– and DLPFC–SAI in the older participants, paired t-tests with Bonferroni correction were applied between TS and SAI (i.e., ISI of N20+2 for M1–SAI; ISI of N20+4 for the DLPFC–SAI).

Next, to compare the degree of modulations between the young and older participants cross-sectionally, we performed independent *t*-tests with Bonferroni correction. Further, to elucidate age-related differences in the M1–SAI and DLPFC–SAI paradigms, we performed Pearson's correlation analyses between age and the following variables: the modulations of MEP–SAI and SAI–TEP in all healthy participants. Moreover, we examined the relationship between modulations of SAI–TEP and cognitive measures in the older participants in M1–SAI or the DLPFC–SAI paradigm. Additionally, we explored the relationship between MEP–SAI modulation and cognitive measures.

In addition, to explore the gender differences for all participants on MEP–SAI and TEP modulations by the M1–SAI, as well as DLPFC–SAI paradigms, independent *t*-tests were performed between female and male participants.

## Results

### MEP–SAI between the young and old healthy participants

Mean intensity (±SE) to induce 1 mV peak-to-peak MEP amplitude of younger and older participants were 80.3 ± 11.5% and 82.8 ± 9.7%, respectively. In the M1–SAI paradigm, the MEP was significantly attenuated compared to TS alone, in the young and older participants by 41.2 ± 8.0% (t_11_ = 4.364, *p* = 0.001) and 32.2 ± 5.9% (t_11_ = 4.977, *p* = 0.0004). However, there was no significant difference between younger and older participants (t_22_ = −0.577, *p* = 0.570) or gender difference (t_22_ = −0.633, *p* = 0.534) in the MEP–SAI.

### Modulation of TEPs by M1–SAI

**Figure 1:**
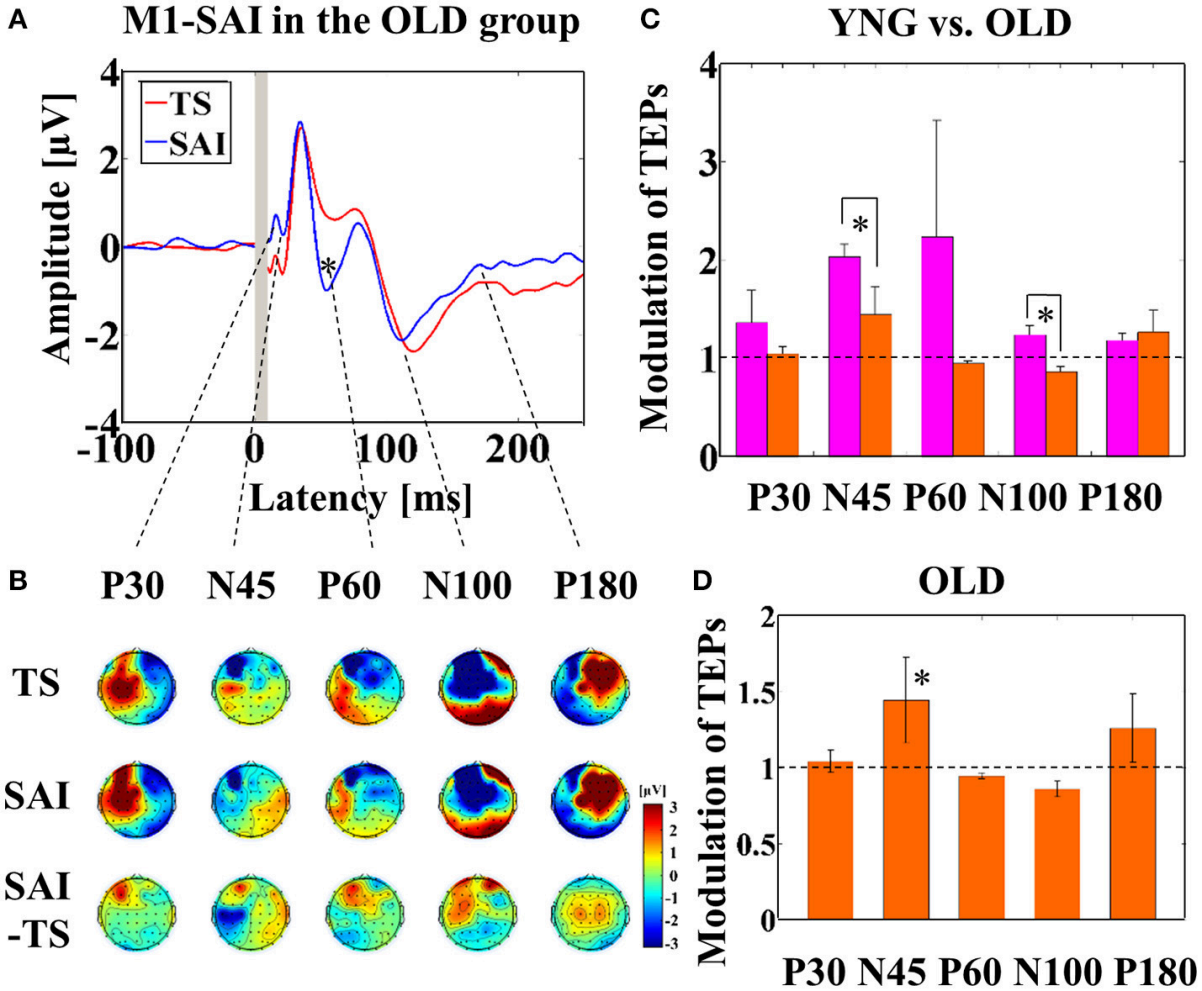
**Result of TEPs in M1–SAI paradigm. (A)** The graph shows the TEP traces with individual somatosensory evoked potentials (SSEPs) subtraction. The graph depicts TEP traces averaged across the older participants for TS and SAI (ISI N20+2 ms) at the left central ROI. TMS was delivered at a time equivalent to 0 ms. **(B)** The illustration shows the EEG topographical plots for conditions of TS, SAI (ISI of N20+2), and the difference between TS and SAI obtained from M1–SAI experiment. Each vertical column depicts the TEP topoplots for P30, N45, P60, N100, and P180 component from left to right, respectively. **(C)** The bar graph shows the group differences between younger (pink bars) and older (orange bars) participants in the modulation of TEPs induced by M1–SAI. The cutoff of the graph is ratio of 1. Older participants demonstrates significantly lower modulation in N45 (t_22_ = 5.485, *p* < 0.0001) and N100 (t_22_ = 3.627, *p* = 0.001) TEPs in this paradigm. **(D)** The bar graph shows the modulation of TEPs within the older participants. The cutoff of the graph is ratio of 1. Older participants indicates significant modulation in N45 TEP (t_11_ = 4.062, *p* = 0.002) by M1–SAI.

Averaged TEP traces and EEG topographical plots by M1–SAI for the older participants are shown in Figures 1A,B (refer to Supplementary Figure 2). For older participants compared to young, independent *t*-tests indicated significantly smaller modulation of N45 TEP (t_22_ = 5.485, *p* < 0.0001; young > old; α–level: 0.01) and N100 TEP (t_22_ = 3.627, *p* = 0.001; young > old) at the left central ROI (Figure 1C). Furthermore, averaged TEP traces without SSEP subtraction in the M1-SAI are shown in a Supplementary Figure 3A. Furthermore, we demonstrated the results of cross-sectional comparisons of SAI–TEP amplitude modulations in M1 between younger and older participants using an amplitude subtraction method in a Supplementary Figure 4A. In this subtraction based analysis, older participants demonstrated a significant increase of N100 TEP amplitude as in the ratio based analysis, but did not show a significant change of N45 TEP.

In addition, within older participants, the M1–SAI paradigm induced a significant increase amplitude on N45 TEP (t_11_ = 4.062, *p* = 0.002; α–level: 0.05/5 = 0.01) at the left central ROI (Figure 1D). Our previously published data contain details on the TEP analyses of the young participants (Noda et al., 2016). Further, there was no gender difference on the modulation of TEPs by M1-SAI paradigm.

### Modulation of TEPs by the DLPFC–SAI

**Figure 2:**
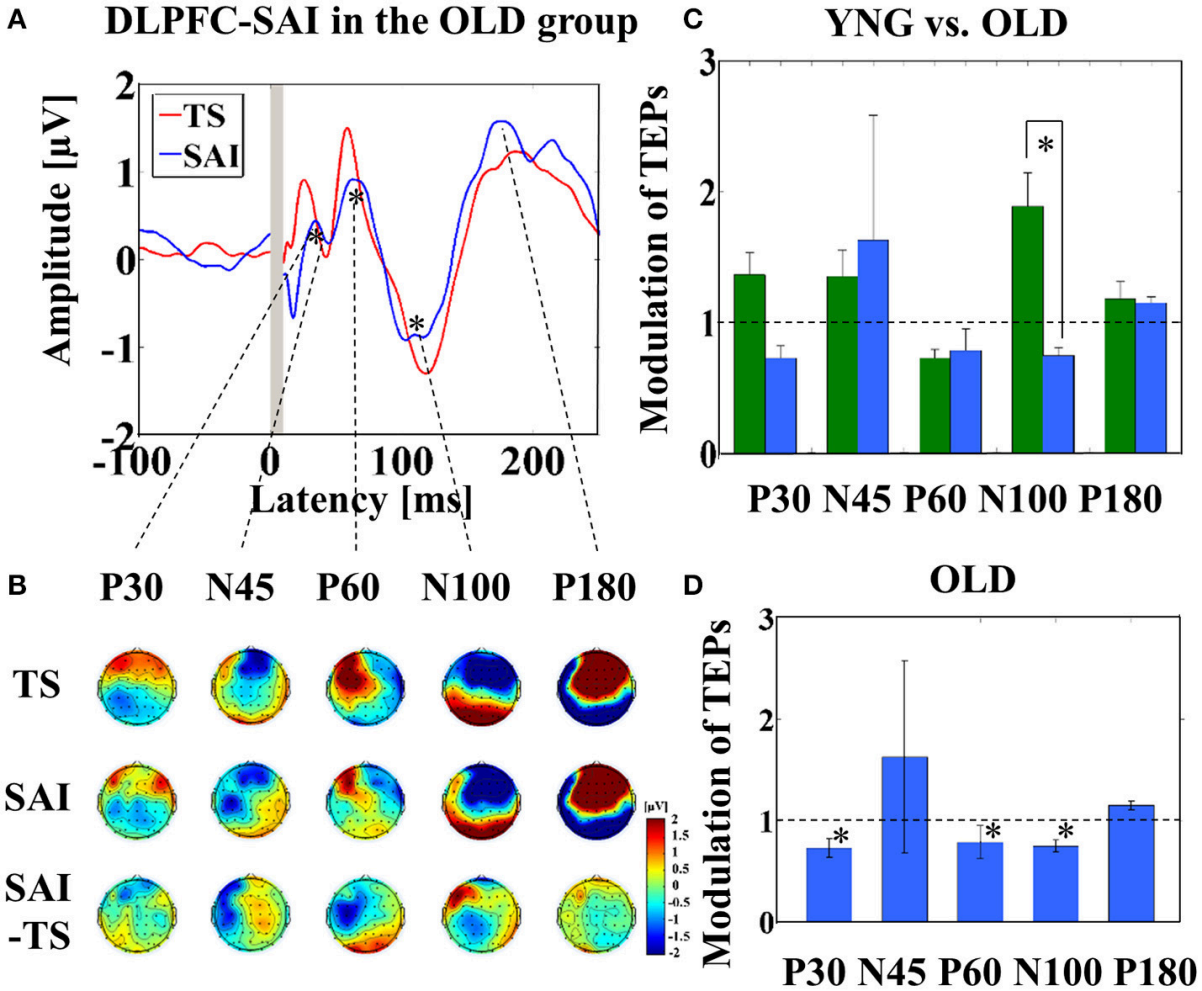
**Result of TEPs in the DLPFC–SAI paradigm**. **(A)** The graph shows the TEP traces with individual SSEPs subtraction. The graph depicts TEP traces averaged across all participants for TS and SAI (ISI N20+4 ms) at the left frontal ROI. **(B)** The illustration shows the EEG topographical plots for TS, SAI (ISI of N20+4), and the difference between TS and SAI obtained from the DLPFC–SAI experiment. Each vertical column depicts the TEP topoplots for P30, N45, P60, N100, and P180 component from left to right, respectively. **(C)** The bar graph shows the group differences between younger (green bars) and older (blue bars) participants in the modulation of TEPs induced by DLPFC–SAI. The cutoff of the graph is ratio of 1. Older participants demonstrates significantly lower modulation in N100 TEP (t_22_ = 2.921, *p* = 0.008) in this paradigm. **(D)** The bar graph shows the modulation of TEPs within the older participants. The cutoff of the graph is ratio of 1. Older participants indicates significant modulation in P30 (t_11_ = 3.204, *p* = 0.008), P60 (t_11_ = 3.165, *p* = 0.009), and N100 (t_11_ = −4.871, *p* < 0.0001) TEPs by DLPFC–SAI. Significant findings are shown with asterisks.

Averaged TEP traces and EEG topographical plots by DLPFC–SAI for the older participants are shown in Figures 2A,B (refer to Supplementary Figure 2). Older individuals showed less modulation in N100 TEP compared to the younger group (t_22_ = 2.921, *p* = 0.008) (Figure 2C). Further, averaged TEP traces without SSEP subtraction in the DLPFC-SAI are shown in a Supplementary Figure 3B. In addition, the results of cross-sectional comparisons of SAI–TEP amplitude modulations in the DLPFC using an amplitude subtraction method are shown in a Supplementary Figure 4B.

Further, within older participants, at the left frontal ROI, P30 (t_11_ = 3.204, *p* = 0.008; α–level: 0.01), P60 (t_11_ = 3.165, *p* = 0.009), and N100 (t_11_ = −4.871, *p* < 0.0001) TEP components were significantly attenuated by the DLPFC–SAI paradigm (Figure 2D). In addition, no gender difference was observed in the modulation of TEPs by DLPFC–SAI paradigm.

### Age-related correlations in M1–SAI and the DLPFC–SAI paradigm

**Figure 3:**
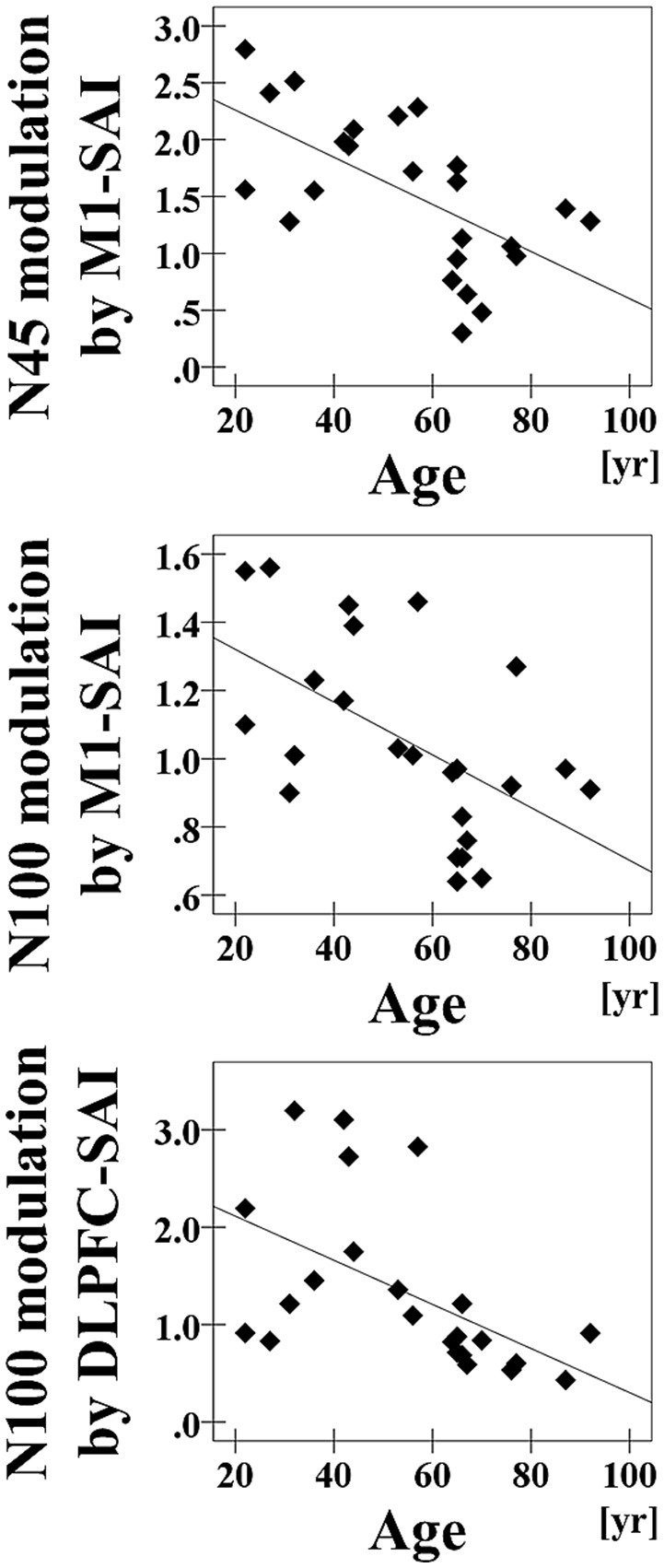
**Age-related correlations**. The results of correlation analyses for all participants between age and the modulation of N45 TEP (*r* = −0.618, *p* = 0.001, *N* = 24) and between age and the modulation of N100 TEP (*r* = −0.548, *p* = 0.006, *N* = 24) at the left central ROI by M1–SAI, and between age and the modulation of N100 TEP (*r* = −0.529, *p* = 0.008, *N* = 24) at the left frontal ROI by the DLPFC–SAI.

Pearson's correlation analyses of all participants demonstrated that increasing age was associated with reduced modulation of N45 TEP (*r* = −0.618, *p* = 0.001, *N* = 24) and N100 TEP (*r* = −0.548, *p* = 0.006, *N* = 24) for SAI at the left central ROI (Figure 3). Further, there was a significant negative correlation between age and the modulation of N100 at the left frontal ROI (*r* = −0.529, *p* = 0.008, *N* = 24) (Figure 3). In addition, the analyses based on a TEP amplitude subtraction method are shown in Supplementary Figure 5.

### Correlation analyses between SAI–TEP modulations and cognitive outcomes in the old healthy participants

**Figure 4:**
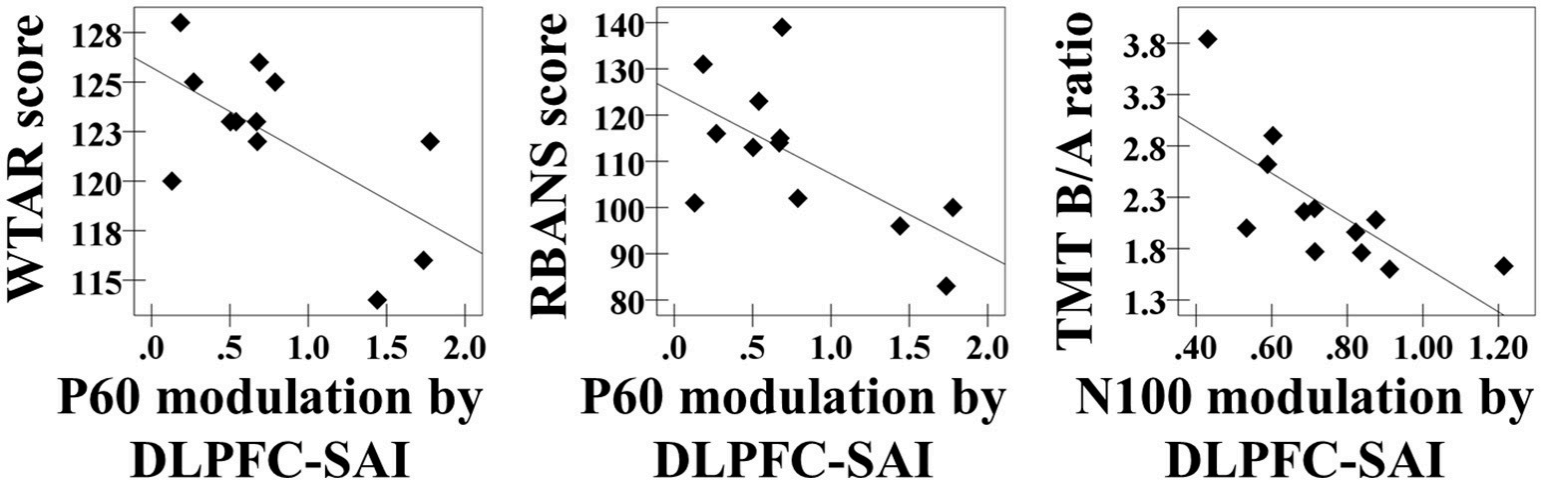
**Cognitive correlations**. The results of cognitive correlations in the DLPFC–SAI paradigm between WTAR standardized score and the modulation of P60 TEP (*r* = −0.632, *p* = 0.028, *N* = 12), between RBANS total scale and the modulation of P60 TEP (*r* = −0.639, *p* = 0.025, *N* = 12), and between ratio of TMT part B to TMT part A and the modulation of N100 TEP (*r* = −0.727, *p* = 0.007, *N* = 12) at the left frontal ROI.

In the DLPFC–SAI paradigm, we observed significant correlations: the WTAR standardized score was negatively correlated with the modulation of P60 TEP (*r* = −0.632, *p* = 0.028, *N* = 12), the RBANS total score was negatively correlated with the modulation of P60 TEP (*r* = −0.639, *p* = 0.025, *N* = 12), and the ratio of TMT part B to part A was negatively correlated with the modulation of N100 TEP (*r* = −0.727, *p* = 0.007, *N* = 12) at the left frontal ROI (Figure 4). Importantly, we did not observe any relationships between cognition and the modulation of TEPs in the M1–SAI paradigm. Further, the analyses based on a subtraction method are shown in a Supplementary Figure 6.

### Correlation analyses between modulations of MEP–SAI and M1–SAI TEPs and DLPFC–SAI TEPs

No significant relationship was observed among these indices in both ratio and subtraction based analyses.

## Discussion

The present study, investigating the SAI effect on TEPs in M1 and the DLPFC for the first time, generated several important findings. First, cross-sectional comparisons between young and older participants in the M1–SAI paradigm revealed that N45 and N100 TEPs were significantly less modulated in older participants compared with younger participants. Second, DLPFC–SAI induced a significant N100 decrease in older participants, compared to younger participants, and further the degree of N100 modulation correlated with age. Third, we observed significant correlations between cognitive function and the modulation of TMS–EEG related markers of SAI (i.e., DLPFC–SAI) in older participants.

The findings of M1–SAI showing lower modulations of N45 and N100 in older participants may demonstrate an age-mediated decline of modulation in both early and late phase of TEPs at M1 Thus, it is possible that GABA_A_ receptor-mediated inhibition of N45 (Ferreri et al., 2011; Premoli et al., 2014) and cholinergic mediated modulation of N100 (Ferreri et al., 2012; Noda et al., 2016) may be declined in an age-dependent manner. More specifically, in the M1–SAI experiment, the modulations of N45 and N100 TEPs were significantly lower in older participants, compared to younger participants, which may indicate that older participants had lower GABA_A_ ergic and cholinergic function. Previous studies have reported that GABA_A_ receptor-mediated short-interval intracortical inhibition is reduced with age (Peinemann et al., 2001; Marneweck et al., 2011; Heise et al., 2013). Another study demonstrated that GABA_B_receptor-mediated inhibition was also reduced with age, as measured with the long-interval intracortical inhibition paradigm (Opie and Semmler, 2014). However, a recent meta-analysis has demonstrated that the effect of aging on MEP from M1-TMS, reflecting GABA receptor-mediated inhibition, are highly variable (Bhandari et al., 2016). Collectively, it seems that central cholinergic activity decays reliably with age (Bartus et al., 1982; Newhouse et al., 1994; Mitsis et al., 2009), whereas the findings of age-related reduction in GABA_A_ receptor-mediated inhibition may be less reliable (Bhandari et al., 2016). Thus, our findings may indicate that M1–SAI paradigm detects cholinergic mediated modulation in a more age-sensitive manner than it detects GABA_A_ receptor-mediated inhibition. In other words, while M1–SAI may induce a similar mechanism of cholinergic mediated modulation on N100 TEP to the DLPFC–SAI, it may not induce the similar pattern of GABA_A_ receptor-mediated effect on the early TEP components compared to the DLPFC–SAI.

The DLPFC–SAI paradigm for both young and older participants demonstrated a significant decrease of P60 TEP at the left frontal ROI. A prior TMS–EEG study utilizing the SAI paradigm in M1 demonstrated attenuation of P60 and N100 TEP amplitudes (Ferreri et al., 2012). Ferreri et al. suggested that this finding may be related to cortico–cortical activation of GABA receptor-mediated inhibition onto the corticospinal neurons (Ferreri et al., 2012). Furthermore, in our previous TMS–EEG study investigating short-interval intracortical inhibition and intracortical facilitation, we observed that an amplitude of P60 was significantly attenuated in the short-interval intra-cortical inhibition paradigm, whereas it was significantly increased in the intracortical facilitation paradigm (Cash et al., 2016), suggesting that P60 may be a useful neurophysiological marker of neural excitability in the DLPFC. Further, our results revealed N100 TEP in the DLPFC–SAI induced robust changes similar to that of the M1–SAI paradigm. However, the direction of the modulation of N100 TEP was opposite between the young and older participants, suggesting that the modulation of N100 by the DLPFC–SAI paradigm may be more sensitive to age compared to M1–SAI. Furthermore, taken together with the finding of age-dependent decline of N100 modulation by DLPFC–SAI, it is possible that N100 modulation by the SAI paradigm is associated with the cholinergic tone as well as GABA receptor-mediated activity in the prefrontal cortex (Young-Bernier et al., 2012; Opie and Semmler, 2014). Moreover, in older participants, DLPFC–SAI induced significantly lower modulations of P30, P60, and N100 TEPs (Figure 2D). Similar to the results of TEP modulations in M1–SAI paradigm, we speculate that partial GABA_A_ receptor-mediated and principally cholinergic mediated modulation may be involved in the DLPFC–SAI neurophysiological responses. However, the modulation on the early TEP components (i.e., N45) by DLPFC–SAI seems to be different in M1–SAI (Figures 1C,2C). This may occur because M1–SAI paradigm can induce both GABA_A_ receptor-mediated and cholinergic mediated functions to some extent, whereas the DLPFC–SAI may detect cholinergic activity in a more specific manner.

We also observed correlations between cognitive measures and inhibitory responses evoked by the DLPFC–SAI paradigm, but not from M1–SAI paradigm. The DLPFC is involved in cognition (Croxson et al., 2011; Yang et al., 2013), and cognitive processes involve cholinergic neurotransmission (Kopelman, 1986; Everitt and Robbins, 1997; Erskine et al., 2004). Therefore, the SAI paradigm in the DLPFC may be associated with cholinergic function in a brain region involved in cognition. Pharmacological studies must be conducted to confirm that DLPFC-SAI is indeed mediated by cholinergic function. More specifically, given the strong correlation between the TMT B/A ratio and N100 modulation, SAI in the DLPFC may be related to executive function.

There are limitations in the present study. First, it is known that the TMS “click” generates an auditory evoked potential, however, we did not use masking noise in this study. This is because the MNS input does not generate an auditory evoked potential—i.e., SAI and TS are matched. Second, in this study, we did not use a magnetic resonance imaging-guided neuronavigation system to localize the DLPFC for each participant. While neuronavigation is more precise in identifying the individual targeting region, the method of approximation using the F5 electrode DLPFC loci has been effective in previous studies (Fitzgerald et al., 2009; Rusjan et al., 2010; Rogasch et al., 2015). To minimize this technical limitation, we applied the ROI-based analysis for TEP data by clustering several electrodes to capture the representative characteristics for both M1 and the DLPFC. Third, cognitive assessment was lacking for the younger participants in this study. Future studies should include the cognitive assessment of younger participants. Fourth, in the absence of a specific pharmacological intervention such as acetylcholinesterase inhibitors, it is not able to demonstrate a causal relationship between cholinergic function and TEPs in the DLPFC–SAI. Thus, the pharmacological intervention study will be needed to confirm the results of DLPFC–SAI. Fifth, in the present study, we analyzed the degree of TEP amplitude modulations by each SAI paradigm in both a ratio and subtraction based approach to confirm the validity of our SAI–TEP analyses. The results demonstrate that there can be somewhat discrepant results depending on the analytical approach taken and caution should be exercised in interpreting TEP modulation data. Notwithstanding this limitation, the main findings persisted across both analyses and suggest that N100 modulation may be the most robust marker of the SAI effect. Finally, the sample size of 12 subjects is relatively small and requires replication in a larger sample

Major neurocognitive disorders such as Alzheimer's disease have been related, at least in part, to loss of cortical cholinergic innervation (Coyle et al., 1983). Modulation of SAI in M1 has been shown to predict long-term response to a cholinesterase inhibitor in Alzheimer's disease (Di Lazzaro et al., 2005b) and have been shown to distinguish between non-amnestic patients and amnestic patients with mild cognitive impairment (Nardone et al., 2012). Thus, the present findings extend the use of the SAI paradigm to the DLPFC in older participants and may be a useful tool to enhance our understanding of the neurophysiological basis of late-life neuropsychiatric disorders.

The present findings, which extend the SAI paradigm in both M1 and the DLPFC to the older participants, have significant potential for future clinical research. In summary, SAI appears reduced in older compared to young individuals. Further, DLPFC–SAI in older participants correlated with executive function. This finding is preliminary and requires further replication and verification. If confirmed in future studies, SAI in the DLPFC may be a potential relevant marker of cholinergic tone. The results of the present study warrant further investigations in populations with cognitive disorders such as Alzheimer's disease and those at risk for Alzheimer's disease such as patients with mild cognitive impairment and late-life depression.

## Author contributions

DB, YN, and ZD were involved in conception and design of the study; YN performed experiments; YN and RZ analyzed data; YN, RFHC, RC, ZD, and DB interpreted results of experiments; YN prepared figures; YN and FB drafted the manuscript; MB, TR, DB, ZD, FB, and RC edited and revised the manuscript; all authors approved final version of manuscript.

## Funding

YN receives postdoctoral fellowship from the Centre for Addiction and Mental Health (CAMH) Foundation. MB receives research support from the Brain and Behavior Research Foundation (Formerly NARSAD) Young Investigator Grant and Schizophrenia Junior Faculty Grant from the CAMH Foundation. RFHC was supported by a Canadian Institutes of Health Research (CIHR)—Dystonia Medical Research Foundation Fellowship award. TR received research support from Brain Canada, Brain and Behavior Research Foundation, Canada Foundation for Innovation, the CIHR, Ontario Ministry of Health and Long-Term Care, Ontario Ministry of Research and Innovation, the US National Institute of Health (NIH), and the W. Garfield Weston Foundation. RC received research support from the CIHR, the Catherine Manson Chair in Movement Disorders, Medtronic Inc. and Merz Pharma. ZD has received research support from the Ontario Mental Health (OMH) Foundation, the CIHR, the Brain and Behavior Research Foundation (Formerly NARSAD), and the Temerty family and Grant family through the CAMH Foundation and the Campbell Institute. ZD received research and equipment in kind support for an investigator–initiated study through Brainsway Inc., and a travel allowance through Merck. ZD has also received speaker funding through Sepracor Inc., and AstraZeneca, served on advisory boards for Hoffmann–La Roche Limited and Merck, and received speaker support from Eli Lilly. DB has received research support from the CIHR, NIH, Brain Canada and the Temerty Family through the CAMH Foundation and the Campbell Research Institute. He receives research support and in-kind equipment support for an investigator-initiated study from Brainsway Ltd. and he is the site principal investigator for three sponsor-initiated studies for Brainsway Ltd. He receives in-kind equipment support from Magventure for an investigator-initiated study. He receives medication supplies for an investigator-initiated trial from Indivior.

### Conflict of interest statement

The authors declare that the research was conducted in the absence of any commercial or financial relationships that could be construed as a potential conflict of interest.

## Supplementary material

Supplementary Figure 1 **The selection of electrodes comprising the left frontal (DLPFC) and central (M1) ROI is demonstrated topographically**.

Supplementary Figure 2 **(A)** TEP traces of M1–SAI in younger participants. TEP trace in red represents TS condition while TEP trace in blue represents SAI condition (ISI N20+2). **(B)** TEP traces of DLPFC–SAI in younger participants. TEP trace in red represents TS condition while TEP trace in purple represents SAI condition (ISI N20+4). Significant TEP modulations by SAI paradigm within a younger group are shown in asterisks.

Supplementary Figure 3 **(A)** M1–SAI in older participants. TEP trace in red represents TS condition while TEP trace (SAI') in blue represents SAI condition (ISI N20+2) without SSEP subtraction. A light blue trace represents SSEP. **(B)** DLPFC–SAI in older participants. TEP trace in red represents TS condition while TEP trace (SAI') in blue represents SAI condition (ISI N20+4) without SSEP subtraction. A light blue trace represents SSEP.

Supplementary Figure 4 **(A)** TEP amplitude differences of M1–SAI between younger and older participants. Compared to younger participants, older participants showed a significant increase of N100 TEP amplitude modulation (SAI – TS) with SAI paradigm (t_22_ = −2.931, *p* = 0.008). However, there was no significant difference on N45 TEP amplitude change between the two groups. **(B)** TEP amplitude differences of DLPFC–SAI between younger and older participants. Compared to younger participants, older participants showed a significant increase of N100 TEP amplitude modulation (SAI – TS) with SAI paradigm (t_22_ = −3.515, *p* = 0.002). Consequently, almost the same results were found in both ratio and subtraction methods in our analyses. Significant findings are shown with asterisks.

Supplementary Figure 5 **Age-related correlations analyzed by a TEP amplitude subtraction method**. There were significant age-related correlations with N45 TEP amplitude modulation at the left central ROI by M1-SAI (*r* = −0.572, *p* = 0.003, *N* = 24), with N100 TEP amplitude modulation at the left central ROI by M1-SAI (*r* = 0.465, *p* = 0.022, *N* = 24), and with N100 TEP amplitude modulation at the left frontal ROI by DLPFC-SAI (*r* = 0.595, *p* = 0.002, *N* = 24).

Supplementary Figure 6 **Cognitive correlations analyzed by a TEP amplitude subtraction method**. There were significant cognitive correlations between WTAR score and P60 TEP amplitude modulation by DLPFC-SAI (*r* = −0.612, *p* = 0.034, *N* = 12), between RBANS score and P60 TEP amplitude modulation by DLPFC-SAI (*r* = −0.744, *p* = 0.005, *N* = 12), and between ratio of TMT B/A and N100 TEP amplitude modulation by DLPFC-SAI (*r* = 0.847, *p* = 0.001, *N* = 12).
