# Supplementary figures and images for: Reduced Prefrontal Short—Latency Afferent Inhibition in Older Adults and Its Relation to Executive Function: A TMS-EEG Study
Source: Front Aging Neurosci. 2017 May 2;9:119. doi: 10.3389/fnagi.2017.00119 (PMC5411436; doi:10.3389/fnagi.2017.00119)

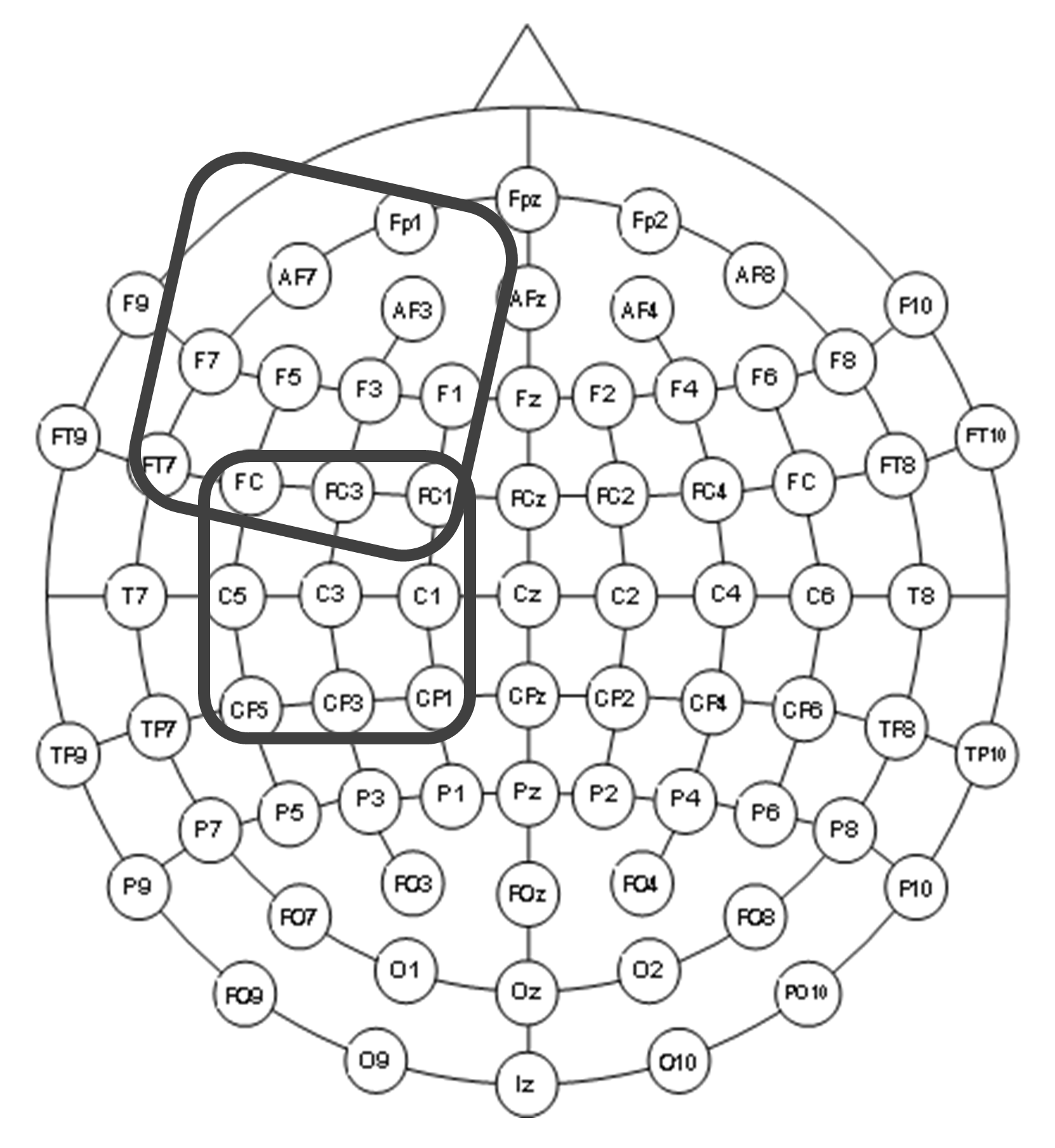

Supplement: Supplementary Figure 1 — The selection of electrodes comprising the left frontal (DLPFC) and central (M1) ROI is demonstrated topographically. [file Image1.TIF]

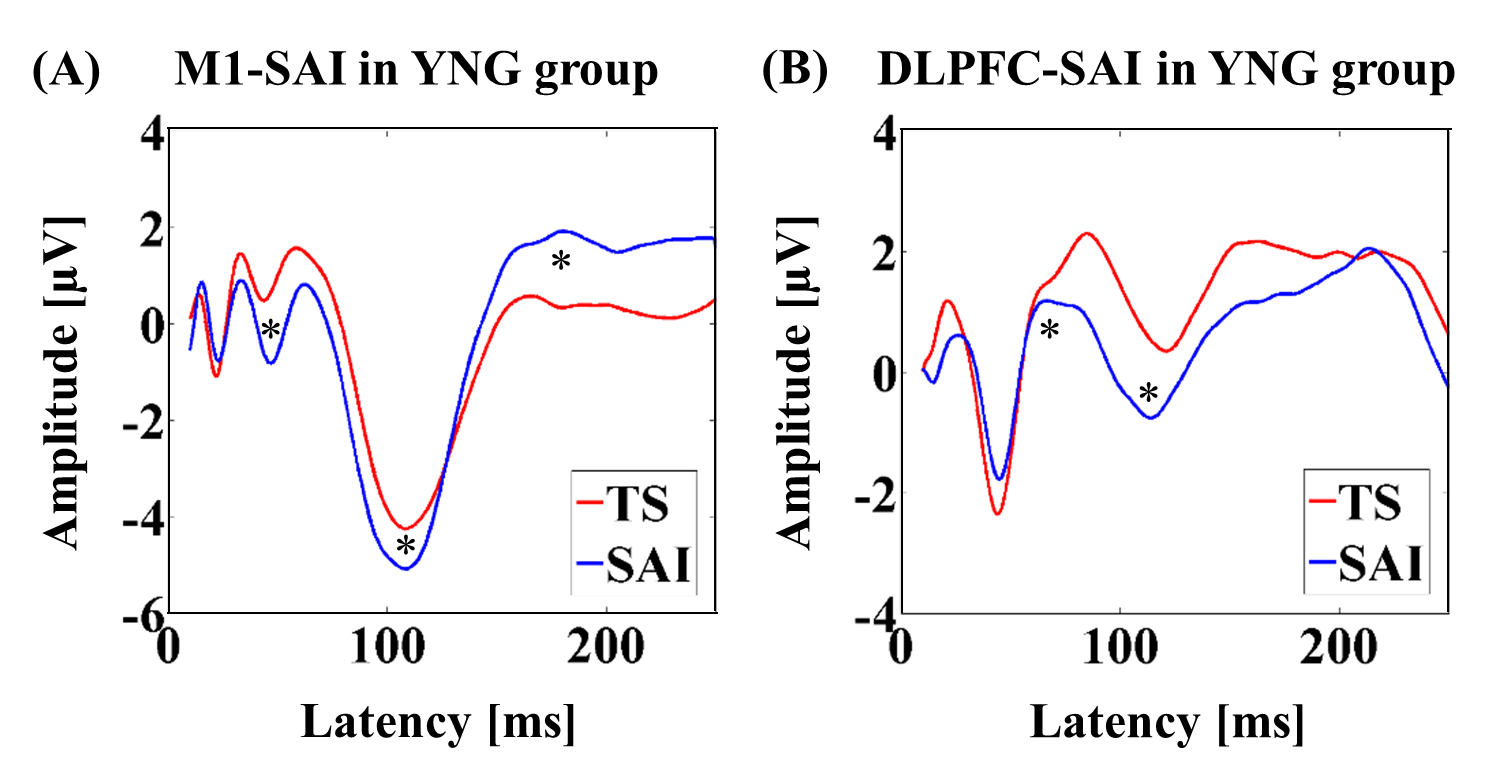

Supplement: Supplementary Figure 2 — (A) TEP traces of M1–SAI in younger participants. TEP trace in red represents TS condition while TEP trace in blue represents SAI condition (ISI N20+2). (B) TEP traces of DLPFC–SAI in younger participants. TEP trace in red represents TS condition while TEP trace in purple represents SAI condition (ISI N20+4). Significant TEP modulations by SAI paradigm within a younger group are shown in asterisks. [file Image2.TIF]

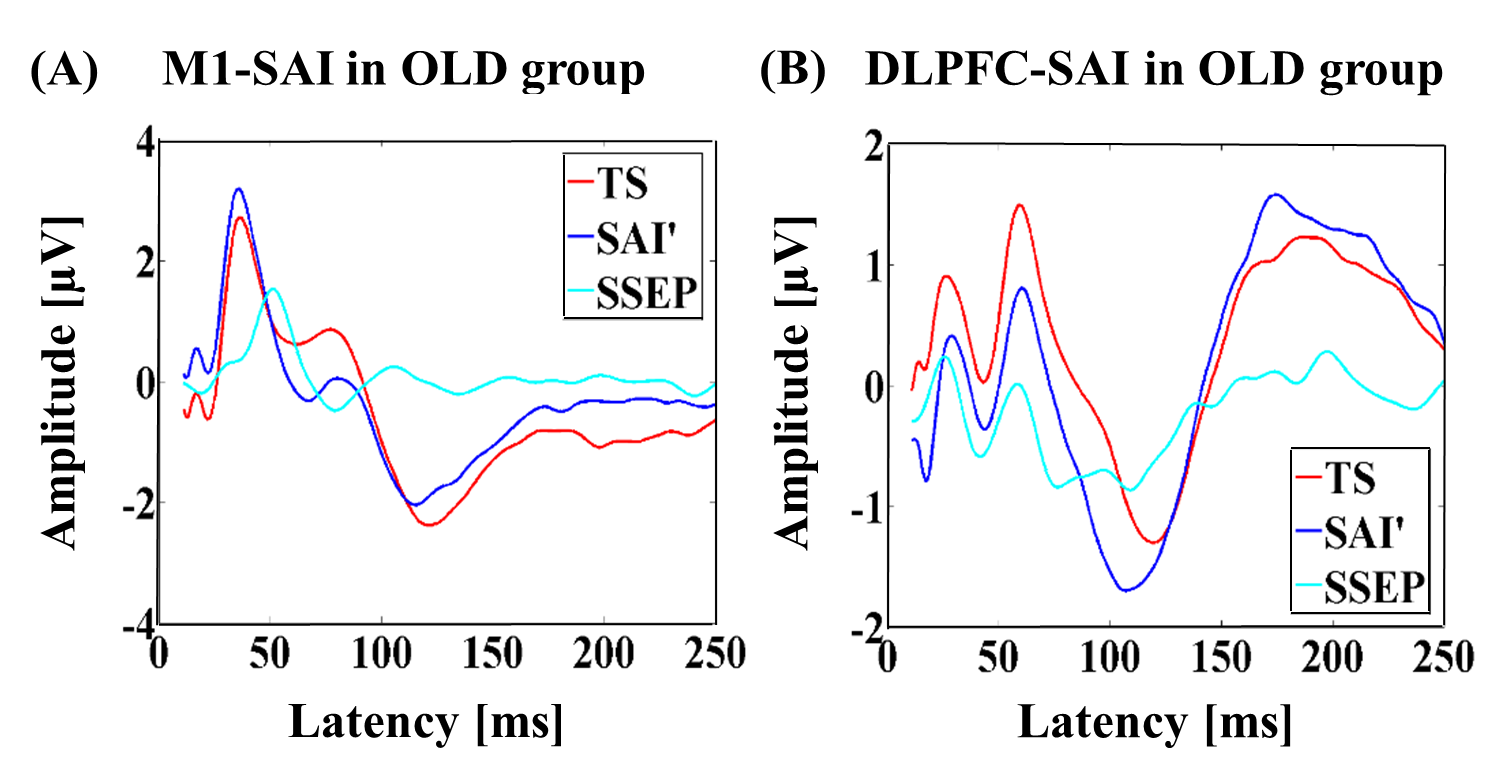

Supplement: Supplementary Figure 3 — (A) M1–SAI in older participants. TEP trace in red represents TS condition while TEP trace (SAI') in blue represents SAI condition (ISI N20+2) without SSEP subtraction. A light blue trace represents SSEP. (B) DLPFC–SAI in older participants. TEP trace in red represents TS condition while TEP trace (SAI') in blue represents SAI condition (ISI N20+4) without SSEP subtraction. A light blue trace represents SSEP. [file Image3.TIF]

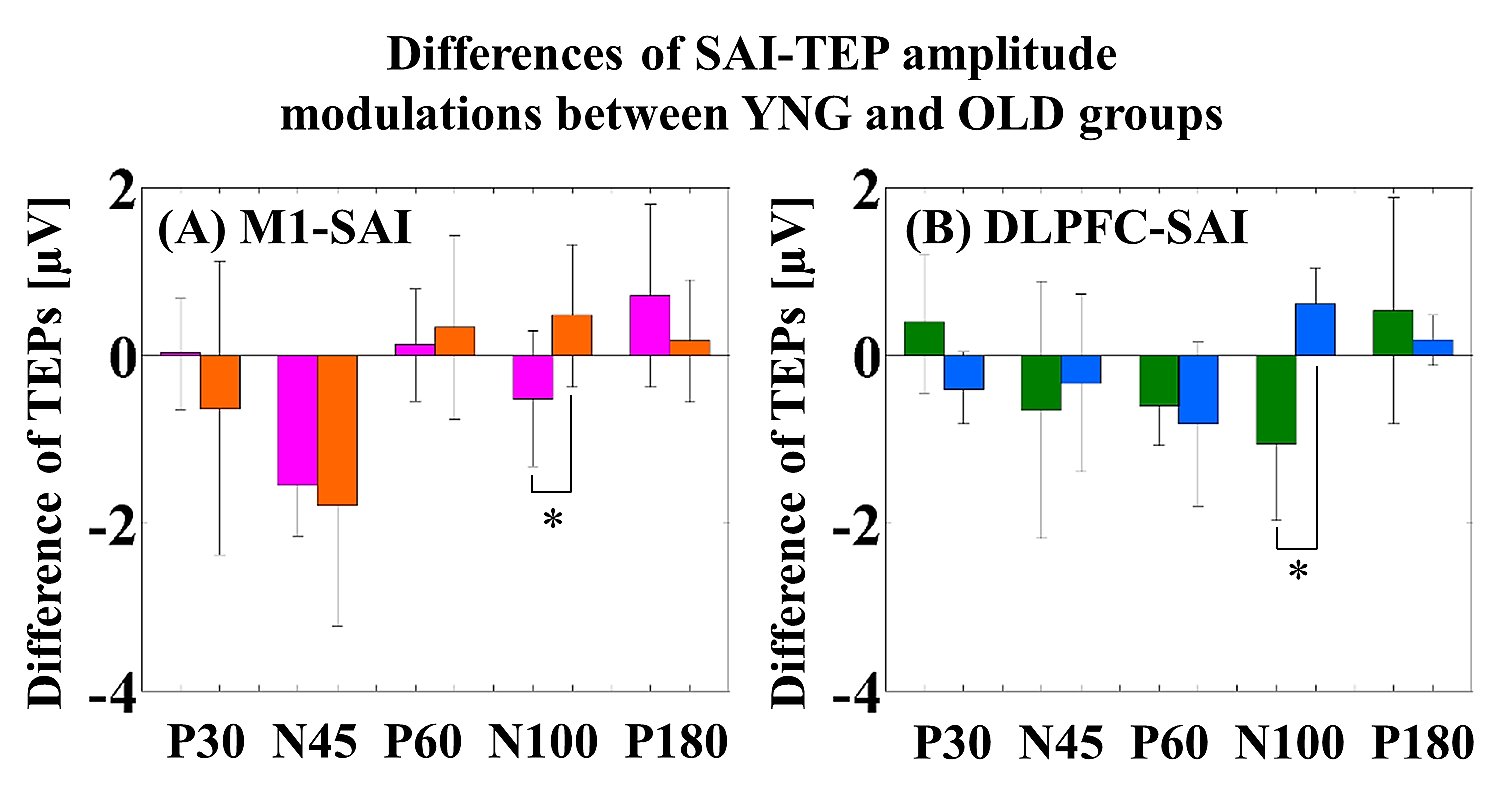

Supplement: Supplementary Figure 4 — (A) TEP amplitude differences of M1–SAI between younger and older participants. Compared to younger participants, older participants showed a significant increase of N100 TEP amplitude modulation (SAI – TS) with SAI paradigm (t22 = −2.931, p = 0.008). However, there was no significant difference on N45 TEP amplitude change between the two groups. (B) TEP amplitude differences of DLPFC–SAI between younger and older participants. Compared to younger participants, older participants showed a significant increase of N100 TEP amplitude modulation (SAI – TS) with SAI paradigm (t22 = −3.515, p = 0.002). Consequently, almost the same results were found in both ratio and subtraction methods in our analyses. Significant findings are shown with asterisks. [file Image4.TIF]

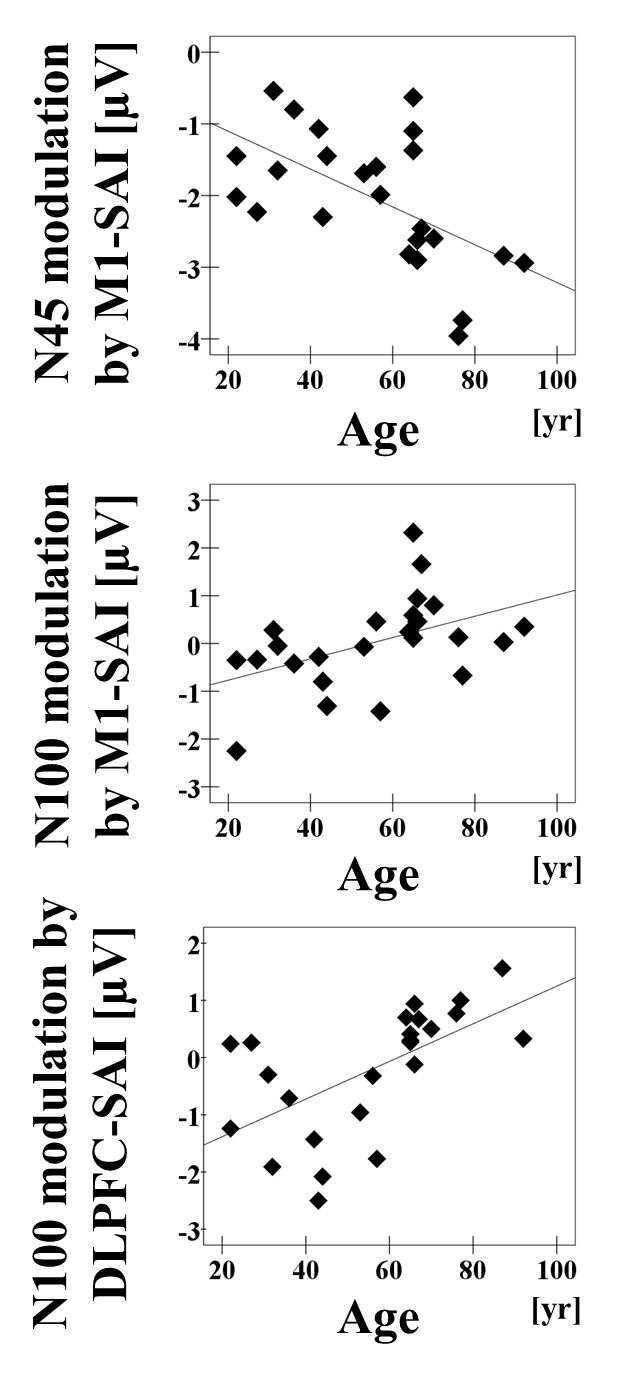

Supplement: Supplementary Figure 5 — Age-related correlations analyzed by a TEP amplitude subtraction method. There were significant age-related correlations with N45 TEP amplitude modulation at the left central ROI by M1-SAI (r = −0.572, p = 0.003, N = 24), with N100 TEP amplitude modulation at the left central ROI by M1-SAI (r = 0.465, p = 0.022, N = 24), and with N100 TEP amplitude modulation at the left frontal ROI by DLPFC-SAI (r = 0.595, p = 0.002, N = 24). [file Image5.TIF]

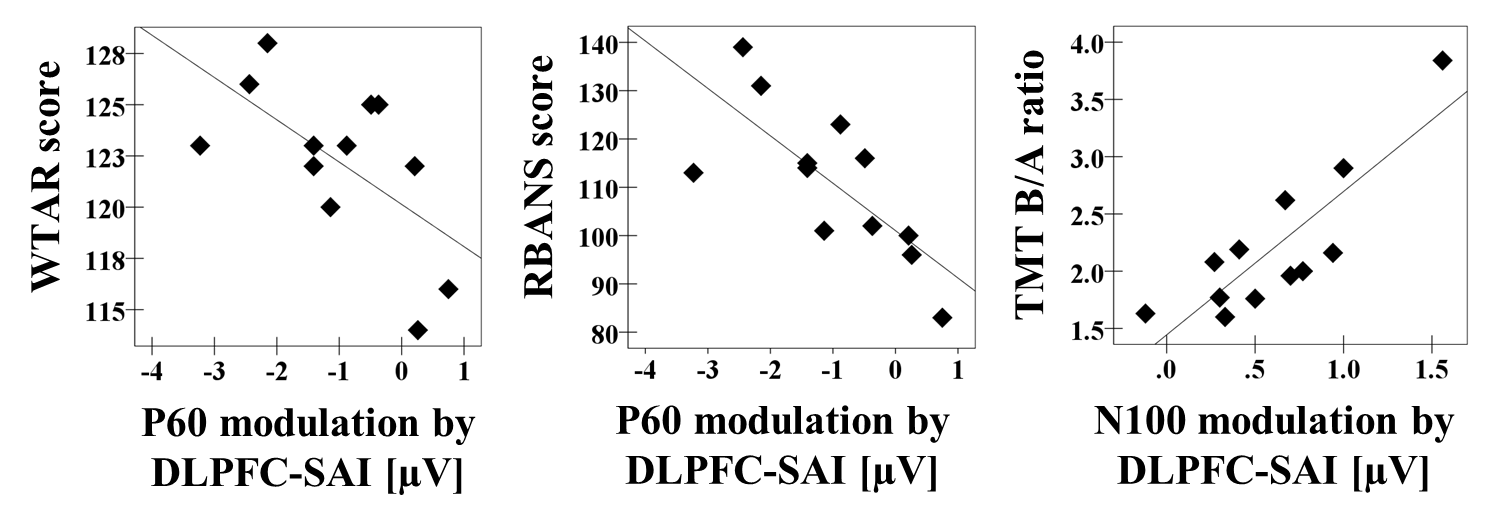

Supplement: Supplementary Figure 6 — Cognitive correlations analyzed by a TEP amplitude subtraction method. There were significant cognitive correlations between WTAR score and P60 TEP amplitude modulation by DLPFC-SAI (r = −0.612, p = 0.034, N = 12), between RBANS score and P60 TEP amplitude modulation by DLPFC-SAI (r = −0.744, p = 0.005, N = 12), and between ratio of TMT B/A and N100 TEP amplitude modulation by DLPFC-SAI (r = 0.847, p = 0.001, N = 12). [file Image6.TIF]
